# Universal alcohol prevention in the workplaces – does it matter?

**DOI:** 10.1177/10519815251320271

**Published:** 2025-03-03

**Authors:** Kristina Sundqvist, Martina Wilson Martinez, Kristina Berglund

**Affiliations:** 1 Department of Psychology, Stockholm University, Stockholm, Sweden; 2 Department of Psychology, University of Gothenburg, Gothenburg, Sweden

**Keywords:** workplace, prevention, preventive measures, alcohol, policy, universal prevention, primary prevention

## Abstract

**Background:**

Harmful alcohol consumption has significant negative implications for the workplace. The workplace offers a strategic opportunity for alcohol prevention due to the substantial time employees spend at work.

**Objective:**

Utilizing a social-ecological framework, this study aims to investigate whether universal alcohol prevention strategies in the workplace are associated with employees’ alcohol-related perceptions or behaviors

**Methods:**

A cross-sectional survey study was conducted in December 2019 using a web-based questionnaire distributed through the Laboratory of Opinion Research Citizen Panel. Participants included 2771 employed adults aged 16–80 in Sweden. Multiple- and logistic regressions were used to investigate significant explanatory factors.

**Results:**

Having alcohol procedures, as well as having received information from a supervisor regarding how to act on concerns, were associated with a higher probability of being comfortable informing a supervisor of concerns. A restrictive alcohol culture was associated with lower threshold for perceived risk-free alcohol consumption. Having an alcohol policy only was not associated with any of the examined alcohol-related perceptions or behaviors when controlling for other factors.

**Conclusions:**

Alcohol preventive measures can significantly influence employee perceptions of responsibility to act on concerns, and the feeling of being comfortable doing so. The findings support the integration of alcohol preventive measures at multiple levels within the workplace, as well as having a restrictive alcohol culture.

## Introduction

Harmful alcohol use has been identified as one of the ten leading risk factors for the burden of disease in all global comparative risk assessments, contributing to over 5% of all deaths.^1,2^ Diseases with well-established associations to harmful alcohol use include cirrhosis,^3^ cancers^4^ and cardiovascular diseases.^5,6^ Therefore, harmful alcohol use is considered a major public health concern. In addition to the risks posed to individuals and society, harmful alcohol consumption has significant negative implications for the workplace. Excessive alcohol use among employees has consistently been associated with negative work-related outcomes such as accidents, injuries, reduced productivity, high turnover rates, increased healthcare costs, absenteeism, and impaired job performance.^7–9^ These consequences highlight the importance for employers to address alcohol-related issues in the workplace. Furthermore, the workplace offers unique opportunities for prevention in countries in which the majority of the adult population is employed and spend a lot of time in the workplace, typically at or in close contact with their colleagues, which maximizes exposure to intervention actions.^10^ Various theoretical models highlight workplace-related risk factors that can influence employee drinking, including workplace culture, job-related stressors, easy access to alcoholic beverages, and social control mechanisms like frequent traveling.^11,12^ Notably, workplace stressors have been found to specifically increase alcohol use before, during, and after work.^12^ Conversely, workplace interventions can target these risk factors, for instance by controlling alcohol availability or setting clear guidelines for alcohol use, in order to reduce employee drinking.

From a public health perspective, prevention efforts should focus not only on individuals with severe alcohol problems but also on those engaging in risky drinking. This approach aligns with the “prevention paradox” theory, which suggests that targeting the population at large, including individuals with slightly elevated risks, can have a greater overall impact on reducing alcohol-related problems compared to focusing solely on high-risk individuals.^13^ Implementing preventive strategies in the workplace can reach a larger group of employees who may engage in risky drinking without apparent alcohol problems, thus addressing a significant proportion of alcohol-related work issues.^14–17^ In addition, targeting all employees provides an opportunity to identify and support individuals without the risk of stigmatization.

Recognizing the potential of the workplace as a setting for alcohol preventive interventions, an increasing number of workplace-based interventions have been developed and evaluated. Research on such interventions has yielded mixed results. Whilst some studies have shown positive outcomes, such as reduced alcohol consumption and improved work engagement,^18–20^ others have found limited effectiveness in changing behaviors.^21^ Selective prevention interventions, such as Brief Interventions, have shown varying results in the workplace.^22,23^ Health promotion measures within personnel care programs have demonstrated some success in reducing risky drinking.^24^ A recent meta-analysis^25^ on both universal and selective interventions concluded that workplace-based alcohol prevention programs demonstrate a statistically significant and beneficial effect in terms of reducing alcohol consumption. This variety in outcome can be attributed to significant heterogeneity in terms of the samples and interventions studied, research methods used, and overall research quality. In addition, implementing prevention interventions in the workplace is complex due to the multilevel structure and the influence of various factors, including workplace culture and characteristics of the workforce, such as age and gender, on alcohol use patterns.

A helpful theory for analyzing preventive interventions involving multiple levels is the social-ecological model. The social-ecological model (SEM) was first suggested by Brofenbrenner^26^ as an ecological systems theory and was later redefined by McLeroy^27^ as a framework for prevention and health promotion. The SEM is a system model that acknowledges that change can occur at any level. The desired outcome of the SEM is patterned behavior, which is seen as being influenced by and having an impact on the social environment.^27^ The SEM has been applied to a range of health issues and prevention programs mainly regarding nutrition, physical activity, smoking, sexual behaviour, and alcohol and substance abuse, usually including four or five analyse-levels.^28,29^

To be applied on alcohol prevention in a workplace context we propose the following four levels:

*Societal level*: refers to the larger societal and cultural contexts that shape individuals’ beliefs and behaviors. This is the context within which the workplace operates, for example broader cultural attitudes towards alcohol use, legal regulations, societal norms, and public health campaigns regarding alcohol.

*Organizational level*: represents external systems that indirectly influence individuals. On this level alcohol prevention strategies could target organizational policies, alcohol-related guidelines, or organizational culture- and norms concerning alcohol.

*Interpersonal level*: represents the immediate environment in which individuals interact. In the workplace, this includes the relationships between colleagues, with supervisors, and within teams. Alcohol prevention in the workplace at this level includes preventive strategies targeting the relational, such as awareness campaigns, training programs, or support networks, that can shape employees’ perceptions, attitudes, and behaviors related to handling alcohol related concerns.

*Intrapersonal level***:** At the individual level, SEM recognizes the characteristics, attitudes, and behaviors of employees. It acknowledges that individuals bring their own beliefs, values, and experiences regarding alcohol use to the workplace. These individual factors can influence how employees perceive and respond to alcohol prevention strategies.

By considering several levels, we believe that the SEM provides a comprehensive framework for understanding the various influences on employee behaviour and attitudes that alcohol preventive interventions can have.

The overall aim of the study was to investigate whether universal alcohol prevention strategies in the workplace were related to 1) employees’ perceived responsibility to act on concerns regarding a colleague's alcohol consumption, 2) whether employees felt comfortable in terms of bringing up concerns regarding a colleagues’ alcohol consumption with a supervisor and 3) employees’ perceptions of what they considered to be a risk-free alcohol consumption.

## Methods

This study was designed as a cross-sectional survey study, in which the data was collected in December 2019 through a web-based questionnaire from the LORE Citizen Panel (https://cors.se/en/lore-en/), a component of the SOM Institute at the University of Gothenburg.

### Participants

Participants in this research project were recruited from the Laboratory of Opinion Research (LORE), a Swedish citizen panel. LORE, affiliated with the University of Gothenburg, which applies web-based questionnaires for data collection. Additional details about LORE and the citizen panel can be found on their website, accessible at https://www.gu.se/en/som-institute/the-swedish-citizen-panel/about-the-swedish-citizen-panel.

For this study, an invitation was sent out in December 2019 to eligible members identified by the LORE Citizen Panel, which included adults between the age of 16–80 who reported being employed. Excluded were participants who were self-employed and others who reported having an alternate primary occupation than employment. The selection process for this study was carried out randomly by the Citizen Panel. Chosen individuals were provided with a concise description of the study, specifying its exclusive focus on employed adults.

All studies conducted through the LORE citizen Panel have received an ethics approval. The approval for this study was given during year 2018 (Exp. 2018-12-11, 1016-18) by the ethics committee the “Central Ethical Review Board” in Gothenburg. All participants in this study gave an informed consent regarding participation. No illiterate participants were involved in the study.

A total of 3302 individuals answered the survey. From those we excluded: 229 participants that reported having an alternative primary occupation than employment (for instance unemployed, a student, or retired), 204 participants that reported being self-employed, and 98 respondents who did not provide a response to the question on employment. Hence, our analytical sample consisted of 2771 participants. See Table 1 for a description of the study population.

**Table 1.** Background variables for the study sample (n = 2771).

	n (%)
*Gender*	
Female	1299 (46.9)
Male	1472 (53.1)
*Age*	
Under 30 years	182 (6.6)
20–39 years	670 (24.2)
40–49 years	760 (27.4)
50–59 years	807 (29.1)
60 years and over	352 (12.7)
*Education*	
Compulsory school	40 (1.4)
Highschool	762 (27.5)
Vocational education	504 (18.2)
University	1465 (52.9)
*Industries*	
Industry/IT/technology	513 (18.5)
Service	521 (18.8)
Health care	370 (13.4)
Construction	197 (7.1)
Transport/logistics	176 (6.4)
Public administration and defence	379 (13.7)
Education	282 (10.2)
Other	315 (11.4)
*Sector*	
Private	1312 (49.9)
The state	389 (14.8)
The region	186 (7.1)
The municipality	546 (20.8)
Other	197 (7.5)
*Number of employees*	
1–49	400 (14.5)
50– 49	818 (29.7)
50–249	628 (22.8)
250– 499	206 (7.5)
500– 100	177 (6.4)
1000 and over	524 (19.0)
*Work position*	
Employed	1958 (71.1)
Human Relations	90 (3.3)
Manager with personnel responsibility	274 (10.0)
Manager without personnel responsibility	202 (7.3)
Executive	152 (5.5)
Other	76 (2.8)

In the socio-ecological model (Bronfenbrenner, 1977), there are several levels that can influence how individuals behave. In our study, we examined variables in the organizational-, interpersonal- and the intrapersonal level.

#### Organizational level

*Alcohol prevention.* The variables regarding universal alcohol prevention measures were: “Is there a written document describing the alcohol policy in your workplace?” [policy], “Are there procedures in your workplace that describe how the employer should handle a case in which an employee has an alcohol problem?” [procedures], and “Have you received any training related to alcohol and/or alcohol-related problems during the time that you’ve been employed at your workplace?” [education]. The response options to the questions listed above were “Yes”, “No” and “I don’t know.

*Alcohol provided by the employer in work-related contexts*. The participants were asked to answer the number of times that the employer had provided the employees alcohol in work-related contexts during the past 12 months. The response options were: Never, once, 2–5 times, 6–10 times, 11–20 times, 21–40 times, more than 40 times. We have merged the variables into the following since there were few numbers in many of the response options: Less than 6 times (indicator variable) and 6 times or more.

#### Interpersonal level

*Information from the manager.* The independent variable that detected the interpersonal level was designed as whether the employee had received information from one's manager regarding how to act on concerns regarding a colleague's alcohol-related issues. The question was “*Have you ever received information from your manager on how to act if you are concerned that a colleague has alcohol-related issues”?* The response options were “Yes”, “No” and “I don’t know”.

#### Intrapersonal level

*Gender, age and education.* The response options for gender included woman, man and non-binary. Since none of the participants responded being non-binary, the gender variable was coded into man (0) as the indicator variable and woman (1). For the variable age, participants could choose to indicate their age within the following age ranges: 18–39 years (indicator variable), 40–59 years, 60 years or older. The following categories for education were: high school, vocational education and university (indicator variable).

### Dependent variables

The study focused on three primary dependent variables.

*Perceived responsibility whether to act on concerns regarding a colleague's alcohol consumption.* The first was an index of questions related to a perceived responsibility to act on concerns regarding a colleague's alcohol consumption.

The index was based on five questions:

1. Do you feel a responsibility to act on concerns (e.g., inform a colleague in the workplace) if your colleague appears to be under the influence of alcohol at work?
2. Do you feel a responsibility to act on concerns if your colleague arrives at work with a hangover about two days per month?
3. Do you feel a responsibility to act on concerns if your colleague appears stressed, irritated or unfocused at work, which you believe is due to excessive alcohol consumption?
4. Do you feel a responsibility to act on concerns if your colleague's work performance deteriorates, which you believe it is due to excessive alcohol consumption?
5. Do you feel a responsibility to act on your concerns when your colleague's health deteriorates, and you believe that it is due to a high excessive alcohol consumption?

Response options for the five questions above were: “Does not apply at all (1), Does not apply very well (2), Applies quite well (3), Applies perfectly (4)”. Each employee's responses to the five questions were summed (a minimum sum of 1 and a maximum sum of 20) and aggregated into one index. We assessed the internal consistency of this index using Cronbach's alpha, ensuring that all items were measuring the same construct reliably. The Cronbach's alpha for the index was 0.816, indication an acceptable internal consistency.^30^

*Being comfortable in terms of bringing up concerns regarding colleagues’ alcohol consumption with a supervisor.* The second dependent variable was based on the question “If you were concerned about a colleague's alcohol consumption, would you feel comfortable expressing your concerns to your manager?” The response options were as follows: “Yes, completely comfortable” (1), “Yes, quite comfortable” (2), “No, not very comfortable” (3), “No, not comfortable at all” (4). We categorized the response option ‘completely comfortable’ as one category (1), and the remaining response options were grouped into the other category (0).

*Perceptions of risk-free alcohol consumption.* The third dependent variable in the study was based on the following question: ‘Risk-free alcohol consumption’ is an alcohol intake that does not entail an increased risk in terms of harmful physical, psychological or social consequences. In your opinion, what is the threshold for risk-free alcohol consumption for a man/woman? A standard glass corresponds to approximately: 1 glass (13 cl) of ‘regular’ wine (alcohol content 12–14%), 1 bottle (33 cl) of strong beer (alcohol content 4.8–5.8%), 1 small glass (4 cl) of spirits (alcohol content 37–40%). Participants were asked to fill in the number of standard glasses they considered to be a risk-free consumption. Half of the participants were asked about risk-free consumption for a woman, and the other half were asked about risk-free consumption for a man.

#### Co-variates

We controlled for the following variables in our analyses. *Work industries*: Industry/IT, service, public, health care, construction, transport, education and other industries, *Work sector:* Private, the state, the region, the municipality, other sectors, *Number of employees in the organization*: 1–9, 10–49, 50–249, 250–499, 500–1000 and > 1000) and *Work position*: Employee, human resources, manager without staff responsibility, manager with staff responsibility, executive and other). All co-variates were dummy-coded. Work industries were categorized according to the Swedish Standard Industrial Classification (SNI), a statistical standard that makes it possible to compare and analyze data, both nationally and internationally and over time.^31^

### Data preparation and statistical methods

In the descriptive statistics, t-test, one way ANOVA and chi-square analyses were used. Effect sizes (eta-squared, Cohens d, and Cramers V) were calculated for the ANOVA; t-tests and chi-square analyses. To investigate significant explanatory factors for the dependent variables, we used multiple regressions and logistic regression. When it came to the threshold for risk-free consumption, we logarithmized the responses prior to conducting multiple regression analyses, as the data was not normally distributed. One extreme value (340 drinks per week as the limit for risk-free alcohol consumption) was removed since it was clearly a measurement error. Subsequently, we merged the responses into a single variable from individuals who answered regarding risk-free consumption concerning women and risk-free consumption concerning men. Our decision to perform a logarithmic transformation reduced skewness and normalized the distribution, thereby enhancing the model's performance and reliability. Additionally, having a single dependent variable for risk-free consumption was advantageous. The multiple regressions we conducted met the assumptions required of a multiple regression (multicollinearity and tolerance). For the independent variables, we combined the response options ‘no’ and ‘don't know’ in the multiple and logistic regressions, as the analyses required it to obtain a statistically functional model. Statistical significance in this study was determined to be < .05. We used SPSS version 29 to perform the statistical calculations.

## Results

Comparisons of frequency and mean between the independent variables on the dependent variables are presented in Table 2.

**Table 2.** Comparisons of the prevalence and means of the independent variables across the three dependent variables. Data is presented as mean (sd) or n (%).

	Willingness to act	*Cohens d/Eta Squared*	Feeling secure to act	*Cramers V*	Risk-free alcohol consumption Men/women	*Cohens d/Eta Squared*
*Gender*						
Male	15.7 ± 3.2***	.221	541 (36.8)	.004	5.9 ± 6.5/ 4.6 ± 3.3***	.231/.314
Female	16.4 ± 2.2		473 (36.4)		5.0 ± 4.5/ 3.8 ± 2.3***	
*Age-groups*						
18–39 years	15.5 ± 3.1	.016	290 (34.0)	.035	5.5 ± 5.5/ 4.6 ± 3.7	.001/.00
40–59 years	16.2 ± 3.0		590 (37.7)		5.1 ± 3.5/ 4.5 ± 3.8	
60 years or more	16.6 ± 2.8		134 (38.1)		5.5 ± 9.4/ 4.0 ± 3.7	
*Education*						
High school	15.9 ± 3.3	.001	316 (39.4)*	.049	5.2 ± 4.2/4.6 ± 5.2	.001/.002
Vocational education	16.1 ± 3.1		194 (38.5)		5.6 ± 8.0/ 4.5 ± 2.9	
University	16.1 ± 2.9		504 (34.4)		5.2 ± 4.6/4.3 ± 2.7	
*Offered alcohol at work*						
Never	16.1 ± 2.9**	.007	545 (38.1)	.028	5.0 ± 6.4/ 4.0 ± 3.1***	.003/.023
One time	15.9 ± 3.1		182 (39.7)		5.2 ± 4.6 / 4.3 ± 3.3	
2–5 times	15.8 ± 3.1		245 (40.0)		5.5 ± 3.4 / 4.9 ± 3.9	
>5 times	15.2 ± 3.2		42 (33.9)		6.2 ± 3.3/ 6.3 ± 6.8	
*Policy*						
Yes	16.3 ± 2.9 ***	.014	628 (41.1) ***	.099	5.1 ± 4.8/ 4.4 ± 3.7**	.001/.007
No	15.8 ± 3.4		123 (34.1)		5.4 ± 3.8/ 5.3 ± 4.7	
I dońt know	15.5 ± 3.0		262 (30.7)		5.4 ± 6.6/ 4.3 ± 3.0	
*Procedures*						
Yes	16.5 ± 2.9***	.037	740 (42.7) ***	.157	5.2 ± 6.0 /4.4 ± 3.6*	.001/.006
No	15.3 ± 3.7		66 (27.6)		5.9 ± 4.0 / 5.4 ± 5.5	
I dońt know	15.2 ± 3.0		207 (26.8)		5.2 ± 3.8 / 4.3 ± 3.0	
*Education*						
Yes	16.8 ± 2.9***	.021	281 (49.1) ***	.116	5.1 ± 3.4/ 4.5 ± 4.1	.001/.004
No	15.7 ± 3.0		709 (36.1)		5.3 ± 5.9/ 4.3 ± 3.1	
I dońt know	15.4 ± 3.1		24 (28.9)		4.5 ± 2.5/ 5.6 ± 8.4	
*Instructions from manager*						
Yes	16.6 ± 2.8***	.029	423 (49.9) ***	.136	5.0 ± 4.0*/ 4.3 ± 4.2	.006/.001
No	15.5 ± 3.1		509 (35.4)		5.2 ± 4.6 / 4.5 ± 3.5	
I dońt know	15.8 ± 3.0		82 (41.6)		6.6 ± 11.5/ 4.1 ± 2.5	

*p < .05.

**p < .01.

***p < .001.

*Perceived responsibility to act on concerns regarding a colleague's alcohol consumption.* The results showed that women had significantly higher scores on the index “perceived responsibility whether to act on concerns regarding a colleague's alcohol consumption” than men (*t*(2675) = −5.67, *d *= 0.22, *p *< .001). There were also differences between different age groups; the older the age group, the more responsibility they felt to act on concern (*F*(2644, 2) = 20.91, *η² *= .016, *p *< .001). The more occasions the participants reported being offered alcohol at work, the less was their perceived responsibility to act on concerns Above all, it was the group offered alcohol more than five times per year that was the least inclined to act (*F*(2521, 3) = 4.10, *η² *= .005, *p *= .007). If one responded affirmatively to the presence of policy (*F*(2643, 2) = 19.43, *η² *= .014, *p *< .001), procedures (*F*(2643, 2) = 51.00, *η² *= .037, *p *< .001), education (*F*(2523, 2) = 26.44, *η² *= .021, *p *< .001) as well as if the manager provided information on how to act on concern about a colleague's alcohol consumption (*F*(2435,2) = 36.63, *η² *= .029, *p *< .001) one felt a higher degree of responsibility to act.

*Being comfortable in terms of bringing up concerns regarding colleagues’ alcohol consumption with a supervisor.* It was fewer than expected among the group of university-educated individuals who felt comfortable to talk to their supervisor (χ^2^ (2, *N *= 2771) = 6.54, *V *= .049, *p *< .05). It was more than expected being comfortable bringing up concerns, among the groups who had a policy (χ^2^ (2, *N *= 2742) = 27.13, *V *= .099, *p *< .001), procedures (χ^2^ (2, *N *= 2742) = 69.57, *V *= .157, *p *< .001), education (χ^2^ (2, *N *= 2618) = 35.10, *V *= .116, *p *< .001) as well as in the group where the manager provided information on how to act if concerned about a colleague's alcohol consumption (χ^2^ (2, *N *= 2481) = 45.83, *V *= .136, *p *< .001).

*Perceptions of risk-free alcohol consumption.* Men had significantly higher tolerance for risk-free drinking, both in relation to men (*t*(1331) = 4.21, *d *= 0.23, *p *< 0.001) and women (*t*(1281) = 5.73, *d *= 0.31, *p *< 0.001). Those who were offered alcohol in the workplace six times or more in the past twelve months had a significantly more tolerant attitude towards risk-free drinking concerning women (*F*(1226,3) = 9.81, *η² *= .023, *p *< .001). Those who didńt have an alcohol policy in the workplace had a more tolerant attitude towards risk-free drinking concerning women (*F*(1280,2) = 4.75, *η² *= .007, *p *< .01). Those who didn't have a procedure regarding alcohol in the workplace had a more tolerant attitude towards risk-free drinking concerning women (*F*(1279,2) = 4.11, *η² *= .006, *p *< .05). Those who were unaware of receiving guidance from their supervisor on how to address concerns about a coworker's alcohol problem had a more tolerant attitude towards risk-free drinking concerning men (*F*(1218,2) = 3.79, *η² *= .006, *p *< .05).

*Perceived responsibility whether to act on concerns regarding a colleague's alcohol consumption.* We conducted a multiple regression to examine which variables contributed most to the perceived responsibility to act (*F*(2422, 30) = 9.62, *p *< .001). When adjusted for the co-variates, female gender (p < .001), older age (40–50 years: *p *= .016; 60 years or more: *p *= .002) having procedures (p < .001) and having received information from a supervisor how to act if concerned about a colleague's alcohol consumption (p < .001) predicted responsibility to act on concerns regarding a colleague's alcohol consumption, see Table 3.

**Table 3.** Linear multiple regression. Dependent variables: Perceived responsibility to act (n = 2423), perceptions of risk-free alcohol consumption (standard glasses) (n = 2383). In the table, logarithmic unstandardized beta coefficients are presented (standard error in parentheses).

	Perceived responsibility to act	*p*	Perceptions of risk-free alcohol consumption	*p*
*Organizational level*				
Policy (yes)	.034 (.146)	.816	−.018 (.035)	.610
Education (yes)	.250 (.164)	.127	.046 (.039)	.247
Procedures (yes)	.960 (.151)	**<**.**001**	.008 (.036)	.661
*Alcohol served in work-related contexts*				
6 or more times (yes)	−.359(.292)	.218	.237(.069)	**<**.**001**
*Interpersonal level*				
Instructions from supervisor (yes)	.539 (.142)	**<**.**001**	−.059(.034)	.084
*Intrapersonal level*		**<**.**001**	−.179 (.031)	
Gender: Female	.710 (128)			**<**.**001**
*Age-groups*				
18–39 years	Ref		Ref	
40–59 years	.324 (.134)	.**016**	−.034(.032)	.292
60 years or more	.629 (.200)	.**002**	−.138 (0.48)	.**004**
*Education*				
University	Ref		Ref	
High school	−.216 (.146)	.139	−.038(.035)	.073
Vocational education	−.058 (.165)	.723	.003 (.040)	.943
Intercept	14.50 (.31)		1.485 (.074)	
N	2423		2384	
R2	.108		.047	
Adj R2	.096		.035	

Bold font represent significant values, *p* < 0.05.

Adjusted for industry, sector, workplace position, and number of employees.

*Perceptions of risk-free alcohol consumption.* We conducted a multiple regression to examine perceptions of risk-free alcohol consumption (*F*(2422, 30) = 9.62, *p *< .001). When adjusted for covariates, female gender (*p *< .001) and older age (60 years or more; *p *= .004), predicted a more conservative stance towards risk-free consumption. If provided alcohol by the employer in the workplace six times or more in the past twelve months, it predicted an increased tolerance for risk-free consumption (*p *< .001), see Table 3.

*Being comfortable in terms of bringing up concerns regarding colleagues’ alcohol consumption with a supervisor.* The adjusted model showed statistical significance (χ^2^ (30, *N *= 2771) = 180.50, *p *< .001) and explained between 7.1% (Cox & Snell R square) and 9.5% (Nagelkerke R square) of variance in the dependent variable and correctly classified 63.6% of cases. To have experience of education about alcohol issues (*p *= .014), to have routines (*p *< .001), and for the manager to have informed about how to proceed if one is concerned that a colleague has alcohol problems (*p *= .019) significantly increased the odds of feeling comfortable speaking with one's manager about other colleagues’ alcohol habits, see Table 4.

**Table 4.** Logistic regression (n = 2465) where the dependent variable is being comfortable in terms of bringing up concerns regarding colleagues’ alcohol consumption with a supervisor.

	Being comfortable bringing up concerns Odds ratio (CI.0.95)
Organizational level	
Policy (yes)	0.871 (0.71–1.07)
Education (yes)	**1.33** (**1.06–1.67)**
Procedures (yes)	**1.78** (**1.44–2.23)**
*Alcohol served in work-related contexts*	
6 or more times (yes)	0.82 (0.53–1.25)
Interpersonal level	
Instructions from supervisor (yes)	**1.27** (**1.04–1.55)**
Intrapersonal level Gender: Female	1.04 (0.87–1.25)
*Age-groups*	
18–39 years	Ref
40–59 years	1.10 (0.90–1.33)
60 years or more	1.20 (0.90–1.58)
*Education*	
University	Ref
High school	1.12 (0.92–1.38)
Vocational education	1.10 (0.87–1.40)

Bold font represent significant values, *p* < 0.05.

Adjusted for industry, sector, workplace position, and number of employees.

## Discussion

This study investigated whether universal alcohol prevention strategies in workplaces were related to the following three alcohol-related employee perceptions or behaviors: 1) employees’ perceived responsibility to act on concerns related to a colleague's alcohol consumption; 2) feeling comfortable in bringing up such concerns with a supervisor, and 3) employees’ perceptions of what they perceived to be a risk-free alcohol consumption. The results showed that reporting having procedures for handling alcohol-related issues was associated with an increased perceived responsibility to act on concerns. Having alcohol procedures, as well as having received information from a supervisor regarding how to act on concerns, were both associated with a higher probability of being comfortable in informing a supervisor of concerns. In addition, reporting having a restrictive alcohol culture was associated with a lower threshold for perceived risk-free alcohol consumption. Reporting having an alcohol policy was not associated with any of the aforementioned alcohol-related perceptions or behaviors when controlling for other factors.

We utilized the social-ecological model, stating that change in one level within an organization, effect other levels as well. For a more in-depth understanding of employee perceptions and behaviors, in this study we considered both an organizational level, an interpersonal level, but also an individual (intrapersonal) level. The results of the study showed that alcohol preventive strategies on several different levels were associated with employee alcohol-related perceptions and behaviors. When it comes to *the organizational level*, the results showed that the likelihood of acting on concern and feeling comfortable doing so increased when there were clear procedures on what to do if concerned about a colleague's alcohol problem. Having received alcohol-related training also mattered in feeling comfortable bringing up concern with a supervisor. However, a policy itself did not seem to be efficient in terms of employee alcohol-related perceptions. These findings indicate that clear instructions about how to handle a situation are more helpful in increasing employees’ perceived responsibilities to act on concerns, than more passive alcohol preventive strategies such as having a policy only. Previous research on workplace alcohol-policies indicated that, compared to a vague and ambiguous policy, stricter and unambiguous alcohol policies reduce undesirable drinking practices that occur just before coming to work, at work, and during breaks.^32^ Hence, it is possible that we would have seen an association between having an alcohol policy and employee alcohol-related perceptions, should the policies have been strict or with clear guidelines.

At the organizational level, we also had a question about the amount of alcohol served in the workplace, a question that we believe may reflect the organization's alcohol culture. The results showed that the more alcohol served in the workplace, the more liberal were the employees’ perception of how much alcohol could be considered risk-free. In relation to the socio-ecological model, this indicates that organizational level decisions, in this case offering alcohol, could potentially impact individual employees’ perceptions of what constitutes risk-free alcohol consumption. Previous research has shown that workplace alcohol availability and workplace norms have direct effects on alcohol problems^33^ and risky drinking,^34^ and that drinking norms significantly predict workplace drinking.^32,35^ This underlines what has been emphasized in the cultural approach to employee drinking behavior,^36^ suggesting that work settings where alcohol is physically or socially available may promote alcohol use among employees.

When it comes to the *interpersonal level* in the socio-ecological model, we examined whether active communication from the supervisor, regarding how an employee should act when concerned about another employee's alcohol consumption, is significant. The results showed that the likelihood increased for both the perceived responsibility to act on concerns and feeling comfortable acting on concerns, when there was active communication from the supervisor. The results highlight the importance of having an active communication with the employees within an organization, with additional procedures and training at the organizational level on how employees should proceed. To our knowledge, there is no previous study that has examine the associations between employee alcohol-related perceptions and active communication from the supervisor. However, there is a body of research showing the importance of supervisor behavior for employee health^37–39^ and psychological safety.^40^ Hence, it is not surprising that active communication with the employees regarding alcohol in the workplace were associated with employee alcohol-related perceptions in our study. The results also show, in accordance with the social-ecological model, that there are *intrapersonal* factors that influence employees’ perception and behaviors as well. Compared to men, women, more frequently than men, reported a perceived responsibility to act on concerns. Furthermore, women had a more restrictive view of what they considered to be a risk-free alcohol consumption. When it comes to responsibility linked to care and concern for others, previous research has generally shown that women historically have had, and perhaps still have, a higher degree of responsibility and take more responsibility.^41^ Additionally, when it comes to attitudes towards alcohol and alcohol consumption, previous research shows a clear difference between men and women, although the gap has narrowed over the past decades as women's and men's alcohol consumption has become increasingly similar.^42^ Despite this, the results from this study indicate a clear difference for what is considered a risk-free level between men and women, with women having a more restrictive view. Regarding the responsibility to act on concerns, there was also a clear difference related to the employees’ age; the results indicated that the probability of a perceived responsibility to act on concerns increased with age. In the oldest age group (60 years and older), the likelihood of having a more restrictive view of what constitutes risk-free alcohol consumption also increased. This age difference could be due to several factors. For instance, research shows that the older a person is, the greater self-assurance in addressing issues that are sensitive.^43^

Summarily, the SEM model illuminates the different levels affecting, and being affected by, employee perceptions and behaviors. Universal preventive strategies may have to be adjusted according to for example gender– or age composition in the workplace. And how well an alcohol policy has the ability to affect employee attitudes may be dependent on strategies on the interpersonal level, for example how well it is communicated to employees by managers.

### Strengths and limitations

This study has limitations, such as being cross-sectional, which prevents us from drawing conclusions in terms of causality. Nevertheless, the results can be considered interesting and therefore encouraged to be explored further.

The societal level was not examined in this study. This level is hardly targeted in any organizational strategies, however; an awareness of the specific societal context of the organization is important when designing prevention interventions, as well as when comparing with outcomes from studies in other cultures. In Sweden, for instance, there is an alcohol monopoly. Moreover, binge drinking is relatively common compared to other European countries whereas everyday drinking is comparatively less common. Swedish employers have a responsibility for the employees’ mental health, which is regulated in the Swedish Work Environment Act. All the aforementioned societal aspects are likely to interact with the preventive strategies within the organizations.

#### Study sample – lore

Limitations of this study was that this study was designed as a cross-sectional study and the study participants were recruited from a Swedish citizen panel. The LORE Citizen panel relies on volunteers who choose to participate in studies, for which one receives invitations. This self-selection can introduce bias, as individuals who opt to join the panel and the study itself may have unique characteristics or interests that differ from the general working Swedish population, which limits the generalizability of the findings of this study. Furthermore, the sample is non-representative since the composition of a citizen panel may not accurately reflect the demographics of the opted targeted population. Certain groups may be overrepresented as well as underrepresented, leading to a sample that is not representative of the wider population. Furthermore, the chosen data collation implies a sampling bias. Although the recruitment process for the LORE citizen panel is through mail, the invitations to the study was sent to participants via e-mail and the data collection was conducted online. Therefore, individuals without internet access or with limited computer literacy may be excluded. Moreover, the data collected through web-based questionnaires rely on participants’ self-reports, which can be subject to response bias. For instance, participants may provide inaccurate or biased information due to social desirability bias, memory recall issues, or misunderstanding of the questions. Consequently, it should also be noted that we do not know whether any preventive strategies were carried out, or rather that the participants reported they were. We find it important that the limitations listed above are taken into consideration when interpreting the results, as they potentially impact findings of the study.

The use of the social-ecological model enhances the study's theoretical framework, providing a comprehensive perspective on the various influences on employee behavior. In addition, research on alcohol preventive strategies in the workplace is scarce, making this study a much needed contribution.

## Conclusion

The study provides valuable insights into the complex relationships between workplace alcohol prevention strategies and employee perceptions, contributing to the understanding of preventive interventions in the context of the social-ecological model.

Alcohol preventive measures such as having procedures for how to handle alcohol-related issues, communicate to employees on how to act on concerns for a colleague, and providing alcohol-related training can significantly influence employee perceptions of responsibility to act on concerns, and the feeling of being comfortable doing so. However, individual factors such as age and gender also appear to impact the willingness to act on concerns as well as the perceived responsibility to act on concerns, which should be considered when implementing preventive measures. Solely having an alcohol policy might not be enough as a preventive strategy, and should be paired with additional strategies, in order to prove efficient.
